# “Part of a cog, of a system; a system that’s broken”: social workers’ experiences of moral injury in England

**DOI:** 10.1080/17482631.2026.2645257

**Published:** 2026-03-18

**Authors:** Amelia Pearson, Janine Owens, Rebecca McPhillips, Paul Clarkson, Eleanor Hinchliffe, Catherine A. Robinson

**Affiliations:** a Division of Nursing, Midwifery & Social Work, University of Manchester, Manchester, UK; b Bradford District Care NHS Foundation Trust, Bradford, UK

**Keywords:** Ethics, moral injury, social work, well-being, workforce, qualitative

## Abstract

**Introduction:**

Moral injury is the long-term effect of the experience of events that violate deeply held moral beliefs. Social workers in adult care settings are tasked with managing morally complex situations and make decisions that impact the lives of service users. Simultaneously, they adhere to their professional ethical code and organisational standards, potentially giving rise to moral injury. Little is known about these experiences. This qualitative descriptive study aimed to explore social workers’ experiences of moral injury.

**Methods:**

Remote interviews were conducted with ten social work professionals, in adult services in England. Data were inductively analysed using reflexive thematic analysis.

**Results:**

Three themes were identified: a) System-related impacts; b) Service-related impacts; c) Individual psychological impacts. Social worker moral injury can arise from efforts to uphold professional values whilst navigating limited resources, interprofessional conflicts, and working within a constrained system. Witnessing dehumanising, low-quality, and impersonal care is a major source of moral injury. This is marked by professional disempowerment, sadness, guilt, betrayal, and helplessness.

**Discussion:**

Recognising these experiences is important. Building psychosocial resilience through formal support is essential for moral repair. Application of the concept for social work, alongside further research, is paramount to adequately support staff.

## Introduction

Guided by professional standards of ethics (e.g., Social Work England), social work is a values-based profession (Reamer, 2013). Adult social work settings (e.g., mental health centres and hospitals), are considered particularly morally complex (Mänttäri-van der Kuip, 2020). Social workers navigate decisions affecting the lives of vulnerable members of society, including older adults, individuals with learning difficulties, physical or sensory impairments, those accessing or needing mental health services, and people with drug or alcohol addiction (Haight et al., 2016; Mänttäri-van der Kuip, 2020). Moral agency can be compromised when social workers attempt to balance the needs of service users, their professional values, the expectations of their organisations, and their personal beliefs (Mänttäri-van der Kuip, 2020).

The term “moral injury” originates from military research (Shay, 2014). No agreed definition exists, but it is often described as exposure to potentially morally injurious experiences (“PMIEs”): “perpetrating, failing to prevent, bearing witness to, or learning about acts that transgress deeply held moral beliefs and expectations” (Litz et al., 2009, p. 700). Features of moral injury include long-lasting and profound psychological harm, emotional reactions, and impaired functioning (Čartolovni et al., 2021). Core features include guilt, shame, existential conflict, and loss of trust in self and others; secondary features include depression, anxiety, anger, re-experiencing moral conflicts, self-harm, and social difficulties (Jinkerson, 2016). Moral injury leads to maladaptive cognitions and beliefs, such as an inability to forgive, demoralisation, moral disorientation, and eroded trust, meaning, and hope (Fleming, 2023).

The construct of moral injury lies on a continuum (Litz & Walker, 2025; Litz et al., 2022; Mewborn et al., 2023). Within this continuum, moral injury is more harmful but less frequently experienced than “moral distress”, “when one knows the right thing to do, but institutional constraints make it nearly impossible to pursue the right course of action” (Jameton, 1984, p. 6). The consequences of moral distress are short-lived and moderate, often featuring moral repair or restoration (Mewborn et al., 2023). Accumulated, unresolved moral distress can manifest as moral injury, termed the “crescendo effect” (Epstein & Hamric, 2009; Riedel et al., 2022). Perceptions about the degree of impact of moral challenges are subjective, with differing effects between individuals (Mewborn et al., 2023). Moral distress in one individual can occur as moral injury in another. Existing research about moral distress in social workers in England omits the sustained and enduring impact of moral injury (Contreras & Adriasola, 2024).

The health and care sector in England faces major challenges, including chronic underfunding, increasing demand, long waiting lists, widening health inequalities, insufficient staffing, and increasing workforce burnout and stress (Glasby et al., 2021; Dunn et al., 2023). These add complexity to the role of social workers, as they attempt to navigate moral and ethical decision-making. Moreover, social care is overlooked compared to frequent NHS reform and reorganisation (Alderwick et al., 2019). A bleak picture portrays the social work workforce in England, with an average sickness rate of 9.9 days, nearly double the general social care workforce, and a turnover rate of 17.2% (Skills for Care, 2024). The well-being and mental health of social workers recently gained traction as an issue in England and the United Kingdom (UK), during and following the coronavirus pandemic (Davies et al., 2025; Ravalier et al., 2023; Ravalier et al., 2023; Stanley et al., 2025). Evidence identifies challenges to social workers’ well-being, specifically, high burnout and stress. However, less is known about their experiences of moral injury, particularly in England (Hall et al., 2022; McEwen et al., 2020).

Most existing evidence around social worker moral injury is conducted in the United States, with a focus on child protection and hospital settings (Fantus et al., 2024; Haight et al., 2017; Thibodeau & Cutforth, 2025; Thibodeau et al., 2024). Issues of workload pressures, managerialism, bureaucracy, and compromised care are identified as causative factors for moral injury. Exploration of risk aversion and ethical stress in Scottish criminal justice social workers suggests that eroded professional and moral integrity precedes moral injury (Fenton & Kelly, 2017). Research in other helping workforces in England (i.e., healthcare, higher education, and veterinary services) recognise moral injury as a workforce issue, identifying a need for psychological support (Denham et al., 2023; French et al., 2022; Hanna et al., 2023; Hegarty et al., 2022; Williamson et al., 2023).

The aim of the present study is to expand this evidence base, by exploring social workers’ experiences of moral injury in England.

## Materials and methods

Moral injury is constructed through subjective experiences. Social workers use a lens of morals and values which centre on upholding human rights, social justice, and dignity. Context for each encounter differs for each social worker. Qualitative research is best suited to this study to garner these experiences, alongside one-to-one in-depth interviews which explore the complexity experienced by each participant.

Recognising that experiences are multiple and subjective, that realities are contextually and socially constructed, and shaped by interpretation this study uses an interpretivist epistemology (Finlay and Ballinger, 2006). A qualitative descriptive methodology was employed to frame the experiences of participants, by describing and representing social workers’ perceptions of moral injury, focusing on the individuals and their unique experiences (Doyle et al., 2019). Narrative one-to-one interviews were identified as the most appropriate method for data collection to generate rich accounts of individual, complex, and subjective experiences (Alharahsheh & Pius, 2020). Narrative interviewing empowers participants, enabling them to explore their experiences, and providing the freedom to tell personal stories from their own perspectives (Anderson & Kirkpatrick, 2016). The researcher facilitated participant storytelling, by probing and questioning to enhance depth, gain insight, clarify, and provide a space for reflection (Yufe et al., 2019). Reflexive thematic analysis (RTA) was employed following data collection. RTA interprets the raw and un-interpreted data, reflecting on the knowledge and positioning of the researcher and represents research findings that do not transform the data beyond recognition, remaining “data-near” (Braun & Clarke, 2024; Braun et al., 2022; Doyle et al., 2019; Sandelowski, 2010).

The research topic was informed by consultation exercises with members of the adult social care workforce for the SECURE study (https://research.manchester.ac.uk/en/projects/a-social-care-based-evaluation-of-covid-19-understanding-workforc).

### Participants

Social media and word-of-mouth were used to recruit a self-selecting convenience sample of ten registered social workers in England. They represented a range of roles, ages, years of service, and settings. Sample size was guided by the concept of information power, as data richness met the research aim, aligning with qualitative descriptive research (Doyle et al., 2019; Malterud et al., 2016).

### Procedure

Participants received a Participant Information Sheet outlining the research aim, details of participation, and right to withdraw. After obtaining written informed consent, interviews were conducted between November 2022 and May 2023 by Author 1 and recorded through Microsoft Teams.

Narrative interviewing (Anderson & Kirkpatrick, 2016) elicited detailed accounts of experiences and views on morally injurious situations. Participants were asked one initial question: *“Thinking about your professional life since you qualified as a social worker, can you tell me about any experiences you have had that clashed with your moral or ethical values?”* Techniques of active listening, probing, and prompting enhanced depth of response, clarified and deepened meaning, and explored the extent and intensity of impact (Anderson & Kirkpatrick, 2016). A brief topic guide informed prompts and probes covering experience of event(s), impact, shifts in views and perceptions, and effects on relationships, coping strategies, and support.

### Data analysis

A university-approved transcription service transcribed the recordings. Transcripts were checked for accuracy and pseudonymised. Employing RTA, Author 1 undertook inductive coding, which was discussed with co-authors to achieve depth and enhance reflexivity (Braun & Clarke, 2024; Braun et al., 2022; Byrne, 2022). The process generated and refined themes and subthemes (see Appendix A). The research team’s diverse backgrounds, spanning methodological expertise and social work practice, shaped positionality and the conduct of research activities. This enabled deep exploration, objectivity, authentication, and contextualisation (Liu & Burnett, 2022).

Quotations illustrate analytical points. Participants are identified by participant identification numbers (e.g., P1). Square brackets indicate anonymisation or explanatory comments. Bracketed ellipses indicate omitted talk.

### Ethics

The University of Manchester proportionate ethics committee confirmed favourable ethical agreement (Ref; 2022-13429-21722).

## Results

Interviews lasted between 38 and 106 minutes. Table I presents characteristics of the ten participants, spanning a range of settings and service user groups.

**Table I.** Participant characteristics.

Participant ID	Gender	Age range	Ethnicity	Role	Primary setting	Experience^a^
P1	Woman	45−54	White British	Newly Qualified Social Worker	Learning disabilities	<6 months
P2	Woman	35−44	White British	Senior Social Worker	Hospital	2−5 years
P3	Woman	55−64	White British	Independent Social Worker	Generic adults	10−15 years
P4	Man	35−44	White British	Senior Social Worker	Long-term health conditions and frailty	2−5 years
P5	Man	55−64	White British	Social Worker	Learning disabilities	6-9 years
P6	Woman	35−44	White Greek	Social Worker	Homelessness and substance misuse	2−5 years
P7	Man	35−44	White British	Practice Supervisor	Generic adults	10−15 years
P8	Woman	25−34	White British	Social Worker	Hospital	2−5 years
P9	Man	45−54	White Irish	Social Worker	Older adults and mental health	>15 years
P10	Woman	45-54	White British	Social Worker	Mental health	>15 years

^a^ Since professional registration.

Three main themes were identified from the data: a) System-related impacts; b) Service-related impacts; c) Individual psychological impacts. Additional illustrative and context-rich data extracts are available in Appendix B.

### System-related impacts

Across all interviews, participants felt constrained by the care system, identifying systemic issues that hindered their ability to uphold their professional values and carry out their role.

#### Under-resourced systems

Participants felt restricted by high caseloads and waiting lists: “you”ve got pieces of work coming out of your ears and you’ve got no chance of getting care; and that’s when it gets difficult, stressful and demoralising” (P4).

Difficulties arose when resource demands were prioritised above service user needs: “It’s more about clearing beds than it is about meeting the needs of the individuals […] one size doesn’t fit all but that’s the only option” (P2). Participants described pressures to increase caseloads at the expense of meaningful interactions: “Your assessments are pounds. The assessments prove how many people that you are supporting within that area, and with that comes funding” (P5).

Local authority social workers felt conflicted between serving the state and advocating for service users: “you’re acting as an agent of the state, and that can be really not in line with your own morals” (P7). Pressures by management to discharge patients to save money violated values of autonomy and quality care, leaving participants questioning “Where is patient care? Where is the duty of care?” (P9). The toll of bureaucratic and restrictive processes within local authorities led one participant to leave their role: “I didn’t like myself because of the support I was giving others. […] I had to get out” (P3).

#### Conflicting beliefs, values, and practices

Participants described feeling in “battle all the time with health colleagues” (P8), eliciting stark imagery of conflict and a struggle between two opposing sides. This was illustrated by healthcare workers reportedly “making decisions before they even meet the person” (P3), rather than careful assessments of the needs and desires of individuals for quality service delivery.

Further concerns were reported about healthcare professionals’ practices and interpretation of assessments (e.g., the Mental Capacity Act 2005) to “oppress people rather than support their independence” (P2). These perceptions arose from the different belief systems between social work and healthcare, creating considerable moral concern. For example, the dismissal of a patient’s final wishes: “All she wanted to do was go home to die. […] The risk-averse nature of health decided that, actually it wasn’t worth the risk. […] That could have been met safely at home” (P2). These differing beliefs strained relationships between social work and healthcare professionals, creating moral distress that can lead to moral injury if perpetuated.

One participant discussed feeling isolated amongst other professional groups, feeling like a “lone voice”, working within “hierarchies of health” (P7). This participant reflected that, for social workers, “your voice is so small”. Perceived “significant power dynamics” (P9) intensified inter-professional conflicts, leaving social workers feeling increasingly isolated and excluded from integrated partnerships.

#### A failing system

Several participants viewed effective services as unachievable. Some experienced shifts in beliefs about the system, once believing “you could bring about change for people by being a part of the system” but social workers “end up being part of a cog, of a system; a system that’s broken” (P7). The metaphor of being “a cog” emphasises playing a small and unappreciated part within a mechanised organisation, lacking individuality or significance, apart from their immediate function of service delivery.

Some participants felt the government was failing to support the system: “until there is the money and the resources to be able to do our job the way that we want to do it, it just won't be possible” (P6). Some expressed concerns that funding allocation neglected adult social care: “There is plenty of money, we just don’t get it” (P2); “The NHS can have as much money as it likes, but if there’s no care in the community, that person’s staying in hospital” (P3). This resulted in feelings of hopelessness about the adult social care system, which “won’t flourish until it’s provided with a bigger budget” (P4). Consequently, participants felt betrayed, exacerbating moral injury.

### Service-related impacts

A major source of moral injury emerged from perceptions that the health and social care system is dehumanising and harmful for service users’ physical and mental health.

#### Delivering dehumanising care

Participants felt that sometimes care was dehumanising, impersonal, and lacking in meaningful outcomes. Perceptions were that services intended to help appeared to cause more harm. Examples included increased health needs and reduced quality of life, often caused by prolonged hospitalisation and inappropriate care home admissions:

Before [hospital] they didn’t have any care and support needs. Then because of all the delays and the pressures sometimes they’ve become people that require long-term residential care because of significant deterioration in their health. […] It’s just like devastating. That person’s whole life has just been taken away from them. (P2)

Social workers strive for person-centred care, focusing on choices and decision-making, by working with clients and building relationships. Healthcare appears more detached, viewing patients through a medical lens to discharge once clinically “treated”. For example, during the coronavirus pandemic, with rapid hospital discharges into care homes: “We've got loads of people, now, probably hundreds, if not thousands of people in care homes, that shouldn't be […] morally, that’s just wrong” (P1). These experiences oppose participants’ motivations for entering the profession: “I came into this job to try and improve people's lives” (P6).

Participants felt helpless watching individuals, originally with few care and support needs, deteriorate. The impact of witnessing suffering creates distress because social workers are trained in a social model of care, viewing individuals holistically and building relationships.

#### De-professionalisation

All participants reported feeling stripped of their ability to generate meaningful change and impact. Participants felt their profession lacked visibility, their skillset was trivialised, and autonomy eroded: “you don’t need a social worker because you’re telling me what it [the decision] is anyway […] I want to share my experience and knowledge and be able to assess people and to meet their needs. That’s what I’m trained to do” (P5).

Here, social work is slowly eroded by systemic pressures and the hierarchy of the medical profession. Being unable to carry out the role for which they are trained resulted in dissatisfaction, frustration, and demotivation: “I do feel more negative about social work, now, than I ever have” (P1).

### Individual psychological impacts

Participants described a range of long-lasting psychological impacts resulting from morally injurious experiences. Many sought peer and professional support to make sense of their experiences.

#### Sadness, guilt, and betrayal

Profound sadness and guilt were prominent when service users’ needs were unmet. Participants felt both responsible for “letting them down” (P5) and betrayed by management for ignoring their concerns, resulting in frustration and anger: “Everybody’s screaming and the people at the top can hear the screaming, and they’re just not doing anything about it” (P8). Some reported depression, anxiety, stress, and sleep difficulties.

Participants described vicarious trauma, where indirect exposure to the trauma of others leads to unfavourable changes in emotional and cognitive functioning: “I’ve completely opened myself to her experience. So, all this trauma that her and her son have experienced, I have fully let that trauma wash over me now. So, I feel every bit of what they feel” (P7).

#### Helplessness

Participants felt disheartened, disempowered, and demoralised: “We’re in it [social work] to make a difference in people's lives and improve people's lives, and when you're not able to do that, it's just quite deflating” (P6). Over time, the inability to uphold values, beliefs and practices leads to moral injury.

Some participants remained committed, valuing their role within the system and potentially reducing moral residue and the development of moral injury: “I have to keep fighting, so to speak, because if I don't, then who else will?” (P10). The metaphor of battle is again invoked through fighting for the rights of individuals.

#### The development of psychosocial resilience

Support from supervisors and colleagues appeared necessary to boost morale and motivation; developing psychosocial resilience: “The only thing that’s keeping me at the minute is […] my really supportive team” (P1).

Peer support was an opportunity to offload thoughts and express feelings with “like-minded” (P6) colleagues, reducing loneliness and offering space for reflection and learning: “they help me, I’ll help them” (P3).

Embedded reflective practice appeared inaccessible: “You’re not given space to use critical reflection. […] To have a forum to discuss things like morals, ethics, having these abilities to talk like this is so important and we just don’t have that” (P7).

Organisational focus on workforce resilience was criticised for reframing systemic issues as worker inadequacies: “It’s just putting more responsibility on us to take care of our well-being and be more resilient. But actually, if the system is making us unwell it’s not really us that should be more resilient, it’s the system that should change” (P2).

## Discussion

The findings of this study provide evidence of social workers’ experiences of moral complexities, conflict, and distress in adult settings in England and their deep and enduring impact, indicative of moral injury. Incompatibilities exist between statutory practice and the reality of under-resourced and risk-averse systems. Social workers experienced sadness, guilt, vicarious trauma, and helplessness, particularly when services were harmful and dehumanising. This can develop into chronic disempowerment, betrayal, cynicism, a loss of hope and trust in the profession and system; impacts that are akin to the development of moral injury. These findings reflect emerging evidence from healthcare contexts (Čartolovni et al., 2021; Rabin et al., 2023).

The focus on insufficient resources, restrictive practices, and inadequate services, supports the notion of a continuum and the “crescendo effect”, where accumulated distress transforms into injury (Epstein & Hamric, 2009; Harper & Karypidou, 2024; Riedel et al., 2022). Unresolved moral residue serves as a new baseline for moral distress, evoking deeper reactions in emerging situations. The findings extend existing knowledge about social worker moral distress, confirming initial feelings of guilt and helplessness, simultaneously identifying enduring effects, such as betrayal, loss of trust, and existential wounding; consistent with moral injury (Contreras & Adriasola, 2024; Jinkerson, 2016).

Resolving moral distress through moral repair and restoration potentially strengthens ethical practice and maximises worker development, leading to personal growth and the prevention of moral injury (Pauly et al., 2012). The findings indicate that workers often lack time and space to address moral conflicts, increasing the likelihood of injury. Some researchers suggest that adequate resources to engage in practices and techniques can help social workers prepare for and recover from morally distressing experiences, through formal interventions like self-reflection and narrative writing (Morley et al., 2021). These practices and techniques individualise moral distress and make it the “problem” of social workers by failing to address the underlying causes of moral conflict, but they can facilitate moral coping and may help prevent the development of moral injury. To effectively support social workers, interventions specifically targeting moral injury need implementing at the system level to address moral conflict and foster moral resilience. Some system level examples include psychoeducational workshops, ethics-focused debriefings, acceptance and commitment therapy, and mindfulness-based interventions (Davis-Bosch et al., 2025). To ensure their appropriateness, feasibility, efficacy, and impact, these interventions require further co-produced research and participant-led development.

The Dimensional Contextual Model of Moral Injury proposes that individuals appraise potentially morally injurious events based on the values transgressed, personal agency, harm severity, extenuating circumstances, and the potential for repair (Griffin et al., 2023). These appraisals shape responses, from acute functional moral distress to prolonged impairing moral injury. Development of moral injury depends on individual appraisal, perhaps explaining variation in professional disempowerment and commitment, highlighting the importance of reflective practice and supervision (Ravalier et al., 2023).

The mismatch between organisational financial needs and service users’ support needs evidences the dual role of social workers; serving both the state and the public, which is a longstanding professional tension (Davies, 1994; Zychlinski et al., 2020). Conflicting demands result in existential and identity crises, including a loss of trust in colleagues, managers, and the system as well as diminished faith in their professional capacity to create positive change (Cahill et al., 2023; Jinkerson, 2016). The need to prioritise risk reduction over service quality and patient choice reflects research from medical social workers in the United States and criminal justice social workers in Scotland (Fenton & Kelly, 2017; Thibodeau et al., 2024). The social model guides social work, and challenges occur when integrating social work priorities within the dominant medical model, this creates frustration and mistrust (Kangasniemi et al., 2022). The hierarchy of medicine undervalues social work competencies, skills, and practices. This fosters defeatism, cynicism and hinders decision-making in hospital-based social work (Fantus et al., 2022; Heenan & Birrell, 2018; Heenan, 2023; Thibodeau et al., 2024).

Managerial prioritisation of resources over personalised care and service user outcomes is consistent with research evidence exploring ethical stress and risk aversion (Fenton & Kelly, 2017). This study extends these perceptions about the complexity of ethical decision-making, when organisations attempt to balance individualised care with system sustainability. It further emphasises the need for improved personalised services, manageable caseloads, and reflective practice to assist with increasing social worker resilience (Glasby et al., 2021).

Underfunding is a longstanding issue for the UK social care sector (Glasby et al., 2021). The King’s Fund (2024, 2025) reports that the NHS is resorting to reducing or cutting services, raising concerns for social care amidst growing demand from an ageing population and rising working-age disability. As a partial resolution, one suggestion from participants in this study was for increased and better allocation of funding across health and social care. This suggestion is somewhat simplistic because it does not address the historical legacy and continuation of austerity measures and funding deficits for health and social care, nor does it address rising older populations with increasing needs. Funding is finite and the tension between growing demand from rising older populations and allocation of services may force local authorities to choose between funding adult social care and other services. This creates a “democratic deficit” with a resulting rise in unmet need (Bosch & Isden, 2023).

“Dehumanisation” of patients can facilitate emotional regulation; workers distancing themselves from patients helps them to regulate their own emotions and cope with institutional demands (Hoogendoorn & Rodríguez, 2023). Whilst this may improve performance and reduce emotional exhaustion in some, it can risk moral injury in others (Bright et al., 2024; Hagerty & Williams, 2022). The dehumanisation of patients is a key and driving phenomenon that underlies care-related moral injury (Hagerty & Williams, 2022). The core principles of social workers are human worth and dignity, non-maleficence, human rights, and social justice (International Federation of Social Workers, 2014). Working with these moral, ethical, and rights-based core principles presupposes that social workers will find experiences of patient dehumanisation morally unsettling and distressing.

Restrictive organisational demands, resource protection, and dehumanisation caused betrayal-based and witnessed-based moral injury, previously identified among social care workers (Harper & Karypidou, 2024). Consistent with other helping roles, social workers felt betrayal by management, marked by frustration, anger, and helplessness (French et al., 2022; Hanna et al., 2023; Hegarty et al., 2022). Participants distrusted their superiors, damaging professional identity and eroding faith in the overall system (Cahill et al., 2023).

### Implications for practice and policy

Tailored training is essential for managers to identify and reduce moral injury at macro and micro levels, whilst helping experienced professionals to make sense of their past and future experiences. Furthermore, the present study explored practising social workers’ experiences, their focus on system challenges and issues, and the struggles to maintain core values within organisations. This raises questions about whether training sufficiently prepares students for the reality of the role of the social worker and the moral dilemmas they may encounter. Increasing emotional and moral preparedness during social work training is essential to develop moral resilience and coping during practice (Kranke et al., 2021; Lentz et al., 2021). The expectations of social work students need careful management, including education about the reality of the care system to reduce work-related impacts.

Social support emerged as a key coping mechanism, facilitating moral repair by fostering togetherness and self-forgiveness (Cahill et al., 2023). Workplace support and enhanced emotional supervision are crucial for preventing and alleviating moral injury, which might in turn reduce role stress and burnout, especially for newly qualified social workers (Tang & Li, 2021). Implementing regular reflective supervision, focussing on ethical debriefing and moral resilience, potentially enhances emotional coping and moral restoration. Educational initiatives, about the social work role, for NHS managers and other professional groups could maximise shared decision-making (Fantus et al., 2024).

### Strengths, limitations and future research

The study is the first to explore experiences of moral injury in social workers delivering services to adults in England, offering novel insights into the challenges faced and the long-lasting impact of these. This research does not attempt to make generalisable conclusions about all experiences, but narrative interviewing generated detailed accounts of individual experiences, allowing deeper exploration and identifying potential workforce issues.

An important methodological limitation is the self-selecting sample, which may over-represent social workers experiencing heightened distress. A challenge to this area of research is the absence of a quantitative measure designed to assess social workers for moral injury before interview. Nonetheless, this research provides a valuable foundation for understanding moral injury in this context, evidencing the importance and identifying avenues for further exploration. Specifically, conceptual clarification between moral distress and injury, comparative research with other professionals, and the development of a measurement to examine prevalence and effects.

This research captures experiences of social workers during the period of recovery from the coronavirus pandemic. Whilst important, longitudinal research is crucial to explore whether these effects persist over time (Jamieson et al., 2023). Following a cohort of social workers over time would provide detailed insight into how moral injury develops and presents, potential cause-and-effect relationships, and long-term effects.

## Conclusion

This study explored experiences of moral injury in social workers in adult settings across England. The findings reveal how moral injury arises from efforts to uphold professional values whilst navigating limited resources, interprofessional conflicts, and working within a system they no longer believe in. Witnessing dehumanising, low-quality, and impersonal care is a major source of moral injury, marked by professional disempowerment, sadness, guilt, and helplessness. Whilst recognising these experiences is important, building psychosocial resilience through formal support is essential for moral repair. The long-term risk of moral injury among social workers will persist until reform of the health and social care system enhances the accessibility and quality of social care with appropriate support for the workforce.

## Data Availability

The qualitative data generated and analysed during the current study are not publicly available because they contain personal data, for example, job roles, place of work, colleague names, family names and circumstances, which could identify the participant. Redacted data are available from the corresponding author on reasonable request.

## Appendix B.

### Themes, subthemes, and supplementary illustrative quotations.

**Table:**

Theme	Subtheme	Illustrative quotation
System-related impacts	Under-resourced system	“The pressure that should be important is the pressure to support people to regain independence and autonomy over their own lives. That’s what I want to feel pressured about… not whether people have been in a certain place for a few days too long.” (P2)
	Conflicting beliefs, values, and practices	“You’re often a lone voice as the social worker. You might be the only social worker present in an MDT setting, but you’re working with all these other health professionals who are educated in the medical model, they exist within these hierarchies of health. […] Your voice is so small within that, especially if there’s a consultant or someone who’s seen as more important.” (P7) “You don’t need a social worker because you’re telling me what [the decision] is anyway. […] I want to be able to go and share my experience and knowledge and be able to assess people and to meet their needs. That’s what I’m trained to do.” (P5)
	A failing system	“I always thought being part of the conversation was the most important thing, and you could bring about change for people by being a part of the system. […] Here I am ten years later and what’s changed? Nothing, and what power do I have to change things? And maybe actually you do have to be external to it all because otherwise you do end up complicit. And I just don’t know if I want to do that anymore.” (P7) “I’m in a very difficult position because if it was in my personal life I would say…I would feel free to say, yeah, it's a horrible kind of place and I completely agree with you. […] But I have to also keep in mind always that I work for [name of council] and I represent them. So, there's only so much I can say to them. […] I can't be one-hundred per cent honest with them.” (P6)
Service-related impacts	Delivering dehumanising care	“He was put into a drop-down bed which wasn’t right for him. It was in a prominently older person’s home so there was no peer support, there were no activities, nothing for him, really. [I watched] his mental health decline with no mental health services involved.” (P3) “You know that somebody doesn’t need a residential placement but they do need the care, and if there’s no care, residential placement is the only other option. It’s demoralising, essentially, because you know that all the evidence suggests that when somebody moves into residential care, you’re disempowering them and you’re essentially speeding up their physical decline, their mental decline, their general wellbeing just deteriorates.” (P4)
	De-professionalisation	“I wanted to be a social worker to be involved in creating positive change, but now I am a social worker I feel like maybe social work isn’t the place where you can actually do that. Maybe it’s more just in actual politics.” (P2)
Individual psychological impacts	Sadness, guilt, and betrayal	“[I] was off sick for six weeks. Not wanting to carry on, not wanting to carry on in social work at all.” (P1) “It is a stressful thing, you’re dealing with people’s lives. That’s when it can start making you unwell. I ended up being off for about eleven weeks […] diagnosed with stress, anxiety and depression.” (P5) “I can’t sleep, I can’t go to bed and turn off, my mind will be going.” (P5)
	Helplessness	“I’m being told to go against my own morals, and I don’t want to do that. […] When they start pushing you, pushing you, pushing you, you can’t cope with it. And there is nowhere to go except to go.” (P7) “It’s another moral injury, or a moral dilemma, that you’re requesting care from a service that really you don’t have much faith in in the first place.” (P4)
	The development of psychosocial resilience	“It's good to know that you have people likeminded that you can talk to and share these thoughts with and you're not alone in it.” (P6) “We just blast out thoughts and feelings. […] If there’s something that I’m struggling with that I don’t understand, then I can reach to them for that. They help me, I’ll help them.” (P3) “[I] preferred the work in safeguarding, but I came back to this team for that team support and the positive impact that that had on me, and I found that I could cope much better than when I was on the other team.” (P2)
